# National Association of Dental Examiners

**Published:** 1885-03

**Authors:** 


NATIONAL ASSOCIATION OF DENTAL EXAMINERS.—The third regular meeting of the National Association of Dental Examiners was held in Tulane Hall, New Orleans, on Tuesday, March 31, 1885.

The State boards of Ohio, Indiana, Illinois, Michigan, Georgia, Louisiana, South Carolina, Kentucky, Mississippi, and Maryland were represented by the following gentlemen:

J. Taft and H. A. Smith, of Ohio; S. T. Kirk, of Indiana; A. W. Harlan and Geo. H. Cushing, of Illinois; J. A. Robinson and A. T. Metcalf, of Michigan; G. W. McElhaney and J. H. Coyle, of Georgia; J. S. Knapp, L. A. Thurber, O. Salomon, and J. R. Walker, of Louisiana; G. F. S. Wright, of South Carolina; A. O. Rawls, of Kentacky; A. A. Dillehay, R. J. Miller, W. T. Martin, and W. H. Marshall, of Mississippi; Richard Grady, of Maryland.

The greatest harmony prevailed in their deliberations, and after the fullest consideration the following resolutions were adopted.

*Resolved*, That this association recommends to all State boards of examiners that registration should be made with both the examing boards and the clerks of the county courts.

*Resolved*, That this association thinks that all examinaons of candidates by State boards should be conducted principally in writing, and that a record of such examinations should be kept by the secretaries of the boards.

*Resolved*, That, in the opinion of this association, a diploma from a reputable dental college should be considered as the only evidence of qualification for those who seek in the future to enter the dental profession, and that we recommend to all State boards to secure, at the earliest practicable moment, the amendment of existing laws so as to attain this end.

*Resolved*, That the appropriation, by any person, of any title or appellation to which he is not justly entitled and by which deception and fraud may be practiced is, in the opinion of this association, highly reprehensible, and should be prohibited by legal enactment.

*Resolved*, That this association deems it undesirable that State examining boards should be composed of gentlemen serving as professors in dental colleges, and recommends to the appointing powers of all States that, so far as may be possible, the places of such professors, when their terms of office expire, be filled by those not holding such positions.

The association adjourned to meet in Minneapolis on the first Tuesday in August, 1885.

GEO. H. CUSHING, *Secretary.*

---

